# Enhancing national audit through addressing the quality improvement capabilities of feedback recipients: a multi-phase intervention development study

**DOI:** 10.1186/s40814-022-01099-9

**Published:** 2022-07-08

**Authors:** Michael Sykes, Elaine O’Halloran, Lucy Mahon, Jenny McSharry, Louise Allan, Richard Thomson, Tracy Finch, Niina Kolehmainen

**Affiliations:** 1 Northumbria University, Newcastle upon Tyne, NE7 7XA UK; 2 School of Psychology, National University of Ireland Galway, Galway, Ireland; 3 University of Exeter, South Cloisters, St. Luke’s Campus, Heavitree Road, Exeter, UK; 4 Newcastle University, Richardson Road, Newcastle upon Tyne, UK

**Keywords:** Audit and feedback, Quality improvement, Intervention development, Implementation

## Abstract

**Background:**

National audits are a common, but variably effective, intervention to improve services. This study aimed to design an intervention to increase the effectiveness of national audit.

**Methods:**

We used interviews, documentary analysis, observations, co-design and stakeholder engagement methods. The intervention was described in an intervention manual and illustrated using a logic model. Phase 1 described the current hospital response to a national audit. Phase 2 identified potential enhancements. Phase 3 developed a strategy to implement the enhancements. Phase 4 explored the feasibility of the intervention alongside the National Audit of Dementia and refined the intervention. Phase 5 adapted the intervention to a second national audit (National Diabetes Audit). Phase 6 explored the feasibility and fidelity of the intervention alongside the National Diabetes Audit and used the findings to further refine the intervention.

**Results:**

The developed intervention is a quality improvement collaborative (QIC), containing virtual educational workshop, virtual outreach for local team leads and virtual facilitation of a learning collaborative delivered after feedback has been received. The QIC aims to support national audit recipients to undertake improvement actions tailored to their local context. The target audience is clinical and clinical governance leaders. We found that actions from national audit were constrained by what the clinical lead perceived they deliver personally, these actions were not aligned to identified influences upon performance. We found that the hospital response could be enhanced by targeting low baseline performance, identifying and addressing influences upon to performance, developing trust and credibility, addressing recipient priorities, presenting meaningful comparisons, developing a conceptual model, involving stakeholders and considering the opportunity cost. Phase 3 found that an educational workshop and outreach strategy could support implementation of the enhancements through developing coherence and cognitive participation. We found feasibility could be increased by revising the content, re-naming the intervention, amending activities to address time commitment, incorporating a more structured analysis of influences, supporting collaboration and developing local feedback mechanisms. Phase 5 found adaptation to a second national audit involved reflecting differences in the clinical topic, context and contractual requirements. We found that the behaviour change techniques identified in the manual were delivered by facilitators. Participants reported positive attitudes towards the intervention and that the intervention was appropriate.

**Conclusions:**

The QIC supports local teams to tailor their actions to local context and develop change commitment. Future work will evaluate the effectiveness of the intervention as an adjunct to the National Diabetes Audit.

## Key messages regarding feasibility

- In order to develop a feasible intervention, we iteratively applied theory, evidence and stakeholder involvement across multiple phases
- We found opportunities to enhance feedback recipients’ quality improvement capabilities through improved informational analysis, the tailored selection of improvement actions and the development of organisational commitment.
- The developed intervention implements theory-, evidence- and stakeholder-informed target behaviours through virtual workshops, virtual outreach and virtual facilitated collaborative meetings.

## Background

National audits are a common form of audit and feedback where participants receive information about their clinical performance over a specific time. Audit and feedback leads to modest improvement [1]. There is evidence and theory (e.g. [1, 2]) describing ways to increase the effectiveness of audit and feedback that have the potential to enhance national audits.

Audit and feedback is a complex intervention that seeks to change the behaviour of feedback recipients [1]. There is a lack of evidence about the best method for developing complex interventions [3], however there are common elements to existing best practice principles (e.g. [3–8]). These elements include: clarity of perspective, the use of evidence, theory and stakeholder involvement and a defined approach to implementation. The use of iterative methods to develop complex interventions is recommended; for example, O’Caithain et al. [3] highlighted that co-design can provide a method through which repeated cycles of assessment, review and refinement involving stakeholders are undertaken. It is important both to specify the content of interventions in order to inform delivery, evaluation, refinement and replication [9] and to describe the intervention development process [10]. The template for intervention description and replication (TIDieR) provides a framework to describe the content of complex interventions [11]. Behaviour change techniques are observable and replicable active components that can be used to describe the content of behaviour change interventions [12].

This paper describes the multi-phase development of an intervention to enhance the effectiveness of national audit through the iterative integration of evidence, theory and stakeholder input. There are approximately 60 national audits in England [13]. We sought to develop enhancements that might be transferable between national audits. During development, the intervention was tested both as an adjunct to the National Audit of Dementia [14] and the National Diabetes Audit [15]. The National Audit of Dementia is undertaken approximately every 2 years by a multi-agency group led by the Royal College of Psychiatrists. The dementia audit is voluntary, although hospitals are required to report on whether they have taken part, and involves manual data collection. The dementia audit describes the care provided to approximately 10,000 patients, as well as staff and carer experience and organisational information (e.g. training, policies). The National Diabetes Audit (NDA) provides feedback describing clinical performance by primary and secondary care teams, including specialist foot care, pregnancy and transition teams. It describes the care provided to approximately 3.6 million patients. The diabetes audit contains both mandatory and voluntary components and both automatic and manual data collection. NDA feedback is given quarterly, annually or two-yearly, depending upon the specific element of diabetes care being audited. Both the dementia and diabetes audits are commissioned nationally on behalf of NHS England and seek to improve care. For both audits, feedback is as an Excel spreadsheet and a national-level report, the dementia audit also provides a hospital level report. Almost all hospitals in England take part in each audit. Intervention development was iterative, with the results of earlier phases informing subsequent phases. To reflect the development method, this paper describes the methods and results for each phase subsequentially.

## Method and results

### Overview

The work was undertaken in six phases: in phase 1, we aimed to develop a rich description of one audit (the National Audit of Dementia) in different hospitals. This description was undertaken to inform the development and testing of enhancements to that audit (phases 2–4). Transferability was considered through later work to adapt the enhancements to a second national audit (the National Diabetes Audit; phase 5) and further exploration of feasibility (phase 6).

**Fig. 1:**
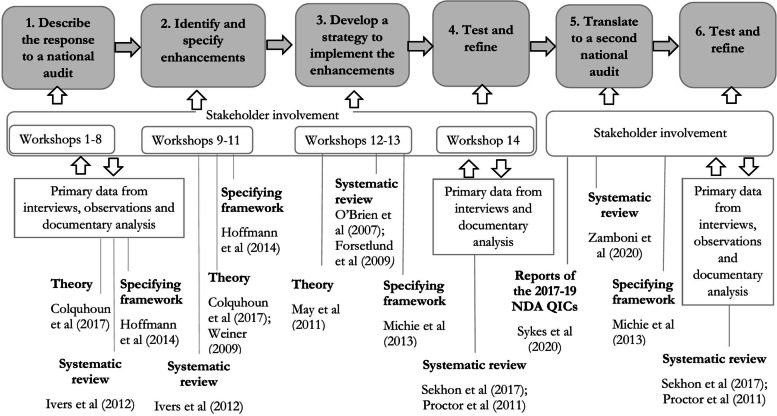
An overview of the study design indicating key inputs to intervention development [17]

Figure 1 represents the study design and illustrates the integration of evidence, theory and stakeholder input across the study’s six phases. Stakeholder analysis [16] identified categories of stakeholders including patients, carers, clinical staff, policy-makers, clinical auditors, regulators, professional bodies, audit provider organisations and researchers. However, to specify which particular stakeholders to involve and to decide and justify a method of involvement, it was necessary to be clear about the reason for involvement [3]. Stakeholder involvement sought to improve feasibility and acceptability through discussion that drew upon diverse perspectives of people anticipated to be involved in potential enhancements. To achieve this goal, stakeholder recruitment involved identifying diverse organisations (organisations that differed in regulator rating and size) and inviting the involvement of clinical leads and clinical audit leads. Carers were sought through two charities (Alzheimers Society and Young at heart) and two organisations that support patient and public involvement in research (Voice North and the Dementias and Neurodegenerative Diseases Research Network). Further stakeholders were identified based upon their role for: the regulator (*n* = 1), relevant professional bodies (*n* = 2), audit provider organisation (*n* = 1), audit commissioner (*n* = 1) and behaviour change researchers (*n* = 3). During phases 1–4, stakeholder involvement was through both a co-design group (involving three carers, three hospital clinical leads and three hospital clinical governance leads) and through an advisory group (*n* = 9; a patient, and representatives from the regulator, relevant professional organisation, national audit provider organisation, national audit commissioner and behaviour change researchers), with the research team providing a conduit between the two groups.

The structure for stakeholder involvement recognised the potential impact of power upon willingness to contribute diverse perspectives (e.g. clinicians in a co-design group, national audit provider, regulator and professional body representatives in advisory different group); co-design group members were supported to provide diverse perspectives through pre-workshop discussions and within-workshop ice-breaker exercise that described and celebrated differences in perspective; facilitation sought to support stakeholders to articulate and explore differences in perspective [18]. The co-design group met face-to-face 14 times (28 h). The advisory group provided input through one whole group meeting and later sub-group meetings. Stakeholder involvement in phases 5 and 6 was through iterative discussions with a group of experts by experience (*n* = 12) and governance groups associated with the National Diabetes Audit.

The dementia audit was selected as it was a national priority [19]. During the study of the dementia audit, Sykes became quality improvement lead for the National Diabetes Audit (NDA). Feedback on this quality improvement work provided the opportunity to adapt the intervention developed alongside the National Audit of Dementia to diabetes care.

Ethical approval for this study was gained both from the Newcastle University Faculty of Medical Sciences and the National University of Ireland (Galway) Ethics Committees.

### Phase 1 method

The aim of phase 1 was to describe what happens when a national audit reaches the hospital. We studied six hospitals in four diverse English National Health Service organisations over a 16-month period. We undertook documentary analysis (*n* = 39), semi-structured interviews (*n* = 32) and 44 h of observations of healthcare workers involved in the response to feedback form the national audit of dementia. Data were analysed using framework analysis and findings were presented iteratively to a co-design group (8 workshops; 16 h) who used them to develop a description of the hospital response to the national audit [18].

### Phase 1 results

We found hospitals staff invested considerable time collecting data and that people collecting data interpreted the current practice and the audit standard differently when assessing compliance. There were delays between data collection and receiving feedback, and when feedback arrived at the hospital it was reviewed by approximately three people, typically two clinical leads and a positional leader with clinical governance responsibility. The clinical leads reviewed the report, often focussing on the national recommendations, and developed a local action plan. There was little evidence that this action plan was informed by local performance or led to the selection of actions aligned to an analysis of influences. The action plan was reviewed at quality assurance committees, where committee members collectively determined a plan to improve performance. The results from phase 1 are reported in detail elsewhere [18].

### Phase 2 method

The aim for phase 2 was to identify and specify enhancements to the national audit. These aims were met through three co-design group workshops (6 h), where inputs to the discussion included the primary data from Phase 1, a reminder of the co-design group members’ stated pre-study views, and presentation of the findings from a systematic review of audit and feedback [1] and theory-informed hypotheses [20]. The facilitator made notes about the discussion on flipcharts during the workshops and in a reflective diary after the workshop. These notes were transcribed after each workshop.

In workshop 9, the co-design group selected a potential target for enhancement using nominal group technique [21]. These potential targets were narrowed by considering feasibility in consultation with the advisory group and research team. In workshop 10, the co-design group discussed the feedback from the advisory group, and further defined the outcome through prompts such as, “what would better action planning lead to?” and “how would you assess whether an action plan was a good one?”.

The research team identified evidence- and theory-informed [1, 20] proposals that might influence the identified outcome (e.g. present loss-framed data; address trust and credibility to increase audit effectiveness) and considered their theoretical coherence. In workshop 11, we presented the proposals to the co-design group and asked: whether they agreed with the proposal, and whether they thought that it might lead to the identified outcome. Group members were then placed into three groups, with each group including a carer, clinical lead and clinical governance lead. The co-design sub-groups completed a task to sort the proposals by categories in the TIDieR framework [11], for example, cluster the proposals according to who could do them, where and when they could be done physically, using post-it notes. Each subgroup presented back to the whole group as part of a discussion that sought to explore differences.

After workshop 11, the notes were transcribed, and the data entered into a table capturing adapted elements of the TIDieR framework (we differentiated between *when* and *how much* and combined *planned* and *actual fidelity)*. The information in the table was then used to describe narratively a series of specified ‘steps’ (Table 1) that captured the aim of the step and who was to do what, when and how. These steps were later amended after phase 3, so as to reflect feedback on influences upon implementation, and amended further following feasibility test in phases 5 and 6.

**Table 1:** A description of each of the seven specified steps

**Step 1:**	
Aims: To address trust and credibility and prepare for action planning	
Who: Clinical lead.	
When: Undertaken before the National audit report is received.	
Preparation step has two parts:	
1. Draft section of report that gives a brief description of:	
a) Source, advisory group representation and external drivers for participation	
b) How data were collected and experienced difficulties with reliable measurement	
c) Refer to later description of triangulation with other data	
2. Prepare for next stage by:	
a) Identify influential members of the specialty and Trust governance groups	
b) Gather Trust board and governance group minutes, quality account, quality strategy and regulator’s (Care Quality Commission (CQC)) report	
c) Identify stakeholder group and arrange meeting(s) to discuss data and improvements	
*Note: This step was amended after phase 3 so as to provide draft brief description and include governance lead familiar with minutes, strategies and regulator reports as a workshop participant.*	
**Step 2**	
Aim: To identify priorities for action from within the hospital feedback.	
Who: Clinical lead and Clinical governance lead	
When: Month 0-1.	
1. Review full data set for potential priorities, where potential priorities are those:	
a) With lower quartile performance	
b) Low absolute performance, where not undertaking target care behaviour might result in significant impact on patient/carer/organisation	
c) For which there is not more robust data that indicates acceptable performance	
2. Identify high performance to celebrate success	
3. Discuss full data set with stakeholder group, targeting on: risks to patient; risks to organisation; triangulation with other data; and successes to be celebrated. Generate a final list of priorities for action with:	
a) Lower quartile performance which is considered unacceptable to stakeholder group	
b) Absolute performance and impact on patient/carer and organisation which is not considered acceptable	
4. Discuss target care behaviours with stakeholder group to identify relationship to other data (e.g. performance, complaints, CQC inspection, length of stay, cost) and organisational priorities (e.g. Trust board, commissioner, CQC).	
**Step 3**	
Aim: To align messages about data to organisational priorities	
Who: Clinical lead and Clinical governance lead	
When: Month 0–1	
1. Review the quality account and minutes from quality committee and organisational board that describe organisational priorities. Consider links to national audit priorities for action	
2. Identify other stakeholders to seek to involve, based upon audit findings and related organisational priorities. Discuss the audit data and the relationship to their priority, whether there is data and/or existing actions that relates to both with these stakeholders.	
*Note: This step was amended after Phase 3 so as to draw upon the clinical governance lead’s knowledge of organisational priorities, rather than review documents.*	
**Step 4**	
Aim: To present prioritised data items in a way that increases motivation to commit organisational resources	
Who: Clinical lead and Clinical governance lead	
When: month 1–2.	
1. Present loss-framed data (e.g. 40% patients did NOT get…)	
2. Present comparison	
3. Identify position compared to own previous performance, national and peer group to be able to give verbal feedback at meeting.	
*Note: This step was amended after phase 3 so as not to recommend loss-framed data due to lack of acceptability.*	
**Step 5**	
Aim: To seek evidence about influences upon performance and potential actions to address barriers	
Who: Participants as described below	
When: Month 1–3	
Seek evidence of influences and actions to address barriers by, for example:	
1. Literature search by hospital librarian of impacts upon performance of target care behaviour	
2. Clinical governance lead reviews Trust data for internal high-performers and national audit data for those beyond the Trust. Ask those identified about what helps performance.	
3. Observe care delivery: Look for possible causes of performance and possible waste (e.g. unnecessary dual data entry) that could be removed to create capacity for change. Observations of care delivery. Findings fed back to clinical lead.	
4. Clinical governance lead: Share findings on noticeboards and ask for reasons via email/anonymous comments. Collate and feedback comments to clinical lead.	
5. Clinical lead: Review list of potential strategies [22]. Ask stakeholder group about barriers and what has been done by others to understand the reasons for current performance (e.g. as part of improvement project, incident review)	
**Step 6**	
Aim: To model the link between barrier, action and organisational priorities	
Who: Clinical lead	
When: Month 3–4.	
Duration: 6 h	
1. Draft logical improvement plan	
2. Discuss draft improvement plan and whether could/should adapt existing actions with service improvement lead, stakeholder group (including deputy director of nursing and influential voices on governance groups) and potential action owners.	
3. Ask whether they agree with the choice of action to address barrier, or whether a different action might be more effective.	
4. Ask potential action owner to take responsibility for completion of the action	
**Step 7**	
Aim: To present to governance group in order to gain approval for the action plan.	
Who: Clinical lead	
When: Month 4–5.	
Describe, verbally and in an accompanying written report:	
1. Data quality;	
2. Prioritisation method and how plan developed;	
3. Successes to celebrate	
4. The logical improvement plans, including relative and loss-framed performance.	
5. The action plan that specifies the target care behaviour, the action to improve detailing: what will be done and the rationale for action; by whom; to whom; by when and how it will be monitored	
*Note: This step was amended after phase 3 so as to provide draft brief description of the method.*	

### Phase 2 results

The co-design group prioritised data collection, feedback and action planning. Consultation with the advisory group and further discussion with the co-design group led to the prioritisation of action planning due to contractual constraints on amending either data collection or feedback delivery.

Facilitated discussion with the co-design group led to them specifying the initial outcome sought from enhancing action planning: an action plan that targets poor performance, describes why not doing well, contains actions which are relevant, actionable, specific, time-bound and measurable. The group further defined each of these terms (Table 7 in Appendix 1).

The research team identified that the following evidence- and theory-informed intervention target behaviours aligned to this outcome: focus on practices with low baseline performance [1]; address recipient priorities; develop trust and credibility in the results; present meaningful comparisons; Use cognitive influences by presenting loss-framed data; identify and address barriers to improved performance; develop a conceptual model of the link between the action and improved care; involve people with control of performance; and consider the opportunity cost of the improvement action [20]. The co-design group considered these proposals, agreeing with each except for the use of loss-framing which they said would not be acceptable. The group said that this lack of acceptability would make it difficult to implement and hinder the implementation of the other enhancements. The research team reviewed the theoretical coherence of the proposed target behaviours, identifying that the development of commitment and informational appraisal to select actions resonated with the theory of organisational readiness for change [23]. The co-design group sorted the intervention targets according to the adapted TIDieR framework criteria. This exercise determined that the target audience was clinical and clinical governance leads. The research team used the notes from the workshop to develop the ‘steps’ described in Table 1; some steps were to be taken simultaneously.

### Phase 3 method

The aim for phase 3 was to develop a strategy to implement the seven steps. The normalisation process theory (NPT) toolkit [24] was used as a heuristic device to explore co-design group members’ reported beliefs about influences upon the implementation of the steps. During workshop 12, each specified step was reviewed individually by co-design group members, and then discussed by the group to surface potential influences upon implementation.

After workshop 12, the research team used the stakeholders’ responses to select and specify the implementation strategy by identifying mechanisms (e.g. coherence) and ingredients (e.g. communal specification) which might affect implementation of each step. We selected a potential type of strategy (educational workshop) based on potential to address the identified ingredients. To develop the content and delivery of the strategy, we drew upon the notes from Workshop 12 and a review describing factors associated with increased effectiveness of the selected strategy (educational workshop [22]). We coded the behaviour change techniques in the draft materials, reviewed consistency, discussed disagreements to seek agreement, and described the intervention in an intervention manual. To review the coherence of the intervention we described it in a logic model which aims to describe the alignment from BCTs, NPT mechanisms, target behavioural outcomes and determinants to patient outcomes. In workshop 13, the research team presented the content and delivery of the intervention to the co-design group. The co-design group suggested amendments. Strategy development in workshop 13 led to the inclusion of an additional strategy (educational outreach), and the consideration of further evidence [25]. We amended the manual and logic model based upon their feedback.

### Phase 3 results

Influences upon the implementation of each step were identified. Looking across steps, we identified the key normalisation process theory (NPT) mechanisms were coherence and cognitive participation and proposed that these may be addressed through an educational workshop and educational outreach (Table 2).

**Table 2:** Influences upon the implementation of each specified step

Key findings and messages from NPT toolkit exercise	
**Step 1: To address trust and credibility and prepare for action planning**	
The semantic differential scale responses indicated the step may not be understand what the step requires of them, may not agree that it should be part of their work, or ‘buy-in’ to the intervention.	
Narrative responses indicated that triangulation would be seen as different; the method could come from existing report; clinical leads may not have the time/capacity to undertake the work (especially in relation to gathering and reading the minutes) but that job planning may be an opportunity but depends upon clinical director support; clinical governance staff may support the step more than clinical lead; may need to be negotiated/arranged well in advance and this may need data, “to hook them in”.	
Techniques to support implementation: 1.1 Goal setting; 1.2 Problem solving; 1.4 Action planning; 4.1 Instruction on how to perform behaviour^a^; 8.7 Graded task^a^; 9.1 Credible source	
**Step 2: To identify priorities for action from within the hospital feedback**	
Responses to the semantic differential scale in the NPT toolkit indicated the step may not be distinguished from current ways of working and key individuals may not drive the step forward.	
Narrative comments included that: There may be different perspectives about what constitutes a priority between the clinical group and the senior leaders; Suggestion to clearly state the aim from prioritising; Suggestion to filter data to short list, rather than review full data set; That those writing the local improvement plan may wish to exclude a target behaviour if they believe they are unable to improve it.	
Techniques to support implementation: 1.1 Goal setting; 1.2 Problem solving; 1.3 Goal setting outcome; 1.4 Action planning; 9.1 Credible source.	
**Step 3: To align message about data to organisational priorities**	
The semantic differential scale responses indicated that individuals may not understand what the step requires of them, may not agree to it becoming part of their work and may not ‘buy-in’ to the intervention.	
Narrative comments included that: it may be difficult to find documents and time to review minutes; those involved may be aware of regulators’ priorities; clinical governance staff may be happy to help; other stakeholders may not engage but that linking to costs (e.g. via length of stay) may support engagement.	
Techniques to support implementation: 1.1 Goal setting; 1.2 Problem solving; 1.4 Action planning; 4.1 Instruction on how to perform behaviour^a^; 5.3 Information about social consequences^a^; 6.1 Demonstrate behaviour^a^; 9.1 Credible source.	
**Step 4: To present prioritised data items in a way that increases motivation to commit organisational resources**	
The semantic differential scale responses indicated that individuals may not understand what the step requires of them, may not agree to it becoming part of their work and may not ‘buy-in’ to the intervention.	
Narrative comments included that: Including positive framing may increase support of key individuals; Comparison should be locally defined, for example, against local hospital; Trust may not allow use of loss-framed data.	
Techniques to support implementation: 1.1 Goal setting; 1.2 Problem solving; 1.4 Action planning; 5.3 Information about social consequences^a^; 9.1 Credible source.	
**Step 5: To seek evidence about barriers and potential actions to address barriers**	
The semantic differential scale responses indicated that individuals may not understand what the step requires of them, may not perceive value in it, may not agree to it becoming part of their work and may not ‘buy-in’ to the intervention.	
Narrative responses indicated that: May not be hospital librarian doing evidence summaries, maybe this should be done by the audit provider; clinical governance team may be pleased to do work to identify high- and low-performing teams and data for triangulation; finding staff time to undertake observation of care may be difficult, although the service improvement team might support this work, but could only do for a few priorities; need to give examples of what ‘waste’ might look like.	
Techniques to support implementation: 1.4 Action planning; 4.1 Instruction on how to perform the behaviour; 6.1 Demonstration of the behaviour; 9.1 Credible source; 12.2 Re-structuring of the social environment; 13.2 Framing/re-framing	
**Step 6: To model the link between barrier, action and organisational priorities**	
The semantic differential scale responses indicated that individuals may not understand what the step requires of them, may not perceive value in it, may not agree to it becoming part of their work and may not continue to support the intervention.	
Narrative responses indicated that: Need to seek agreement from action owners and know what to do if they do not agree.	
Techniques to support implementation: 1.2 Problem solving; 1.4 Action planning; 1.6 Discrepancy between current behaviour and goal^a^; 2.5 Monitoring of outcomes of behaviour without feedback4.1 Instruction on how to perform the behaviour; 6.1 Demonstration of the behaviour; 8.1 Behavioural practice^a^; 9.1 Credible source; 12.2 Re-structuring of the social environment.	
**Step 7: To present to governance group in order to gain approval for the action plan.**	
The semantic differential scale responses indicated that individuals may not understand what the step requires of them, may not agree to it becoming part of their work and may not ‘buy-in’ to the intervention.	
Narrative responses indicated that the participant may only be given a couple of minutes to present at the committee.	
Techniques to support implementation: 1.1 Goal setting; 1.2 Problem solving; 1.4 Action planning; 9.1 Credible source.	

^a^Behaviour Change Technique not included in the Phase 6 manual

The research team also drew upon existing evidence that educational workshops with high attendance, a mixture of interactive and didactic content and that make the target behaviours less complex may be more effective [21]. MS drafted the educational workshop by developing content that addressed the identified ingredients for each step. The draft materials were presented to the research team who proposed amendments to the manual; for example, to avoid social comparison that might undermine the implementation of the target behaviours by removing the description that the target behaviours were not undertaken at the phase 1 study sites. The research team agreed the coherence of the association between NPT mechanism and BCT ingredient. The amended materials were presented to the co-design group to consider face validity. The co-design group proposed further amendments; for example, to amend the workshop booklet to prompt participants to capture tasks and to group the list of potential improvement actions [22] so as to reduce participant burden. The intervention manual was amended in response to this feedback. The group agreed with the proposal to call the intervention, ‘logical improvement planning’.

### Phase 4 method

The aim for phase 4 was to refine the intervention based upon an exploration of fidelity, feasibility, acceptability and appropriateness [9, 26]. We delivered the educational workshop to the target audience (clinical leads and clinical governance lead) at two hospitals within one NHS organisation. Semi-structured interviews explored fidelity of enactment, the acceptability and appropriateness of the enhancements and the acceptability and feasibility of the implementation strategy. The data were analysed using thematic analysis [27]. The analysed findings were presented to the co-design group, the research team and members of the advisory group. The co-design group were asked both to describe their views on whether and how the intervention should be amended and later to comment on proposed changes identified by the research team and members of the advisory group. The intervention was amended based upon their feedback.

### Phase 4 results

We delivered the intervention to four healthcare workers (three clinical leads and one clinical governance lead) in September 2019. We took notes (Appendix 2) and interviewed two clinical leads in March 2020. We sought to interview the other attendees and other potential participants involved in the organisational response to the national audit. One potential participant was willing to discuss the work but did not consent for use of the data. This discussion was used to sense-check findings from the earlier interviewees. Further interviews were prevented by the hospital’s pandemic response.

In exploring fidelity of receipt, participants were able to describe intervention content relating to the identification of opportunities for improvement and the need for work to explore influences; link performance to local priorities; use comparators; reflect existing workstreams. There was evidence for fidelity of enactment and acceptability (Table 3).

**Table 3:** Evidence for fidelity of enactment and acceptability

**Fidelity of enactment**	**Review performance and prioritise** “And so we were into this [the gaps in care] and link performance to priorities. We talked about that, that there is now a high priority on getting dementia.”
	**Select comparator** [Interviewer prompted about the use of comparators] “Yes, yes, now I remember. Exactly. So currently we do not feel we are in a position to compare ourselves to a top 10% because some of the numbers are really low.”
	**Review existing workstreams** “we’ve looked at the audit and we’ve identified gaps in our strategy and we’ve since added those things into our strategy…So some of the gaps were identification of delirium, so we are now going to have 4AT score done on all over 65s who are coming into hospital, whether they’re coming for a medical or a surgical reason, and that will then be rolled out to other admission areas like medical admission area and surgical admission area and other areas. But initially, our focus is towards emergency department.”
	**Identify influences upon performance** “One of the other aspects of strategy, when we looked at the dementia audit, was that we had a training issue. Staff were not trained on dementia, Tier 1 training.”
	**Select strategy aligned to influence** “Staff were not trained on dementia, Tier 1 training. And now we have that as a mandatory field in the electronic service record. So everybody from a cleaner, porter to the chief executive officer will have to be trained.”
**Acceptability**	**Affective attitude, coherence and opportunity cost** “Maybe I’m not reading my emails completely and trying to understand it, but I went into the meeting with some scepticism. What am I going to learn here? But obviously, to my mind, it linked to quality improvement straight away rather than auditing and how this- And so I found it a useful challenge to how we were thinking.” “And it’s basically educating QI process into the audit. So why the audit is done and what should you do with the audit and planning another cycle? What needs to be changed? Yes, those two hours or four hours, were well spent.”
	**Perceived effectiveness** “I think the workshop, in a way, for us gave us a nutshell of how we did in the audit overall. You picked up the domains of where we did well and where we didn’t do well. You gave an overview like this of how to go on about it. So, it was a… Each aspect was looked at in depth, in a way. So, you made us think about the local challenges … to take the next step forward. Even though you didn’t say the right way forward, you made us [see] the right things which would be useful for the trust in a way with the right action plan. So, I think overall it was very useful. Interviewer: Should I have said the things you should be doing? Interviewee: I don’t think you should. So, I think the way you phrased it or led it is- Because obviously each trust is different and unique.”
	**Burden** “The workshops, as I said, maybe half a day. So, just the time could’ve been cut short. Telephone calls, maybe give a bit more time.” And later expanded on this to add, “it’s a national project with the reputation of the trust at stake, but if you’re given such a responsibility there should be a dedicated time.” The same participant later said that the time commitment would be acceptable if there was recognised, and suggested that this could be in the job plan agreed with their manager, “it’s not the time. So, as with anything… It’s the recognition. Exactly. Again, you can see it’s the frustration of not being recognised for so many other things and this comes on the top of one more”.

Interview participants described potential enhancements to the intervention: to create opportunities to learn from others and adding in a monitoring mechanism to the content; to provide those leading the work with feedback about the impact of their improvement strategies:

“What works in other places? And how do we get there? So we have our data already now. We look at where it works well and what have they done to address these things. And then we start implementing those in ours. So that’s how standard approach works…you would need another audit cycle here at PDSA (plan-do-study-act) to prove which of those interventions have actually worked” (Interviewee 2)

In addition to the interview data, notes made during intervention delivery were presented to the co-design group in workshop 14. The group identified that the participants’ preconceptions about the intervention (e.g. “I went into the meeting with some scepticism”) may have adversely affected participants’ initial buy-in. They proposed changing the name of the intervention. They agreed that new components to gain feedback about the effectiveness of the intervention and support sharing between teams may further enhance the intervention. We changed the name to ‘Quality Improvement Collaborative’ to address participants’ reported prior misunderstanding about the aim of the intervention and to reflect the use of shared learning. In response to findings that participants moved quickly to selecting solutions, it was agreed that additional content should take them through ‘within-workshop’ activity to undertake a more structured analysis of influences upon behaviour and an exercise to identify stakeholders. The group felt that bringing more of the work into the workshop, rather than training within the workshop for later independent completion, may also help reduce the reported time burden.

The logic model and materials were amended to reflect the changes agreed with the co-design group: In response to finding a narrow range of influences drawn upon by participants, we introduced an exercise to select influences using the theoretical domains framework [28]. In response to the finding that participants jumped to stakeholders associated with solutions, we introduced a new exercise to identify stakeholders, consider their influence and interest [16] and identify strategies to increase their interest and influence through exploration of their priorities and use of comparators. New content was developed to support participants to develop feedback mechanisms and monitor changes over time. We sought to increase collaboration between teams, through asking teams to describe their plans, facilitating group discussion and introducing monthly virtual facilitated meetings to describe experience, support both group problem solving and resource sharing (e.g. previously produced patient leaflets, guidelines or business cases). The addition of peer description of strategies and apparent burden of the ‘expert recommendations for implementing change’ (ERIC; [22]) exercise led to the exercise to review 64 potential strategies being removed.

### Phase 5 method

The aim for phase 5 was to adapt the intervention to a different national audit. The National Diabetes Audit provides feedback describing the care provided to approximately 3.6 million people by more than 6000 general practices and over 100 specialist diabetes teams in England and Wales [15]. From 2017 to 2019, the National Diabetes Audit (NDA) sought to increase improvement through delivery of four quality improvement collaboratives. These collaboratives, led by MS, used an adapted Breakthrough Series method [29] by supporting clinical teams to engage stakeholders, set aims, select priorities, identify and align actions and monitoring impact. Face-to-face workshops and teleconferences sought to implement the Breakthrough Series practices. For each of the four 2017–2019 QICs, there was an end-of-collaborative workshop for teams to present their work, describe what they had learned and give both written and verbal feedback on the approach. Approximately 160 participants from 70 teams joined these face-to-face meetings, with each team providing feedback on their work. The NDA QIC delivery team collated and discussed participant feedback [30]. Recognition of the need to amend the NDA QIC created the opportunity to adapt the dementia QIC to support diabetes audit feedback recipients.

Adaptation of the intervention from dementia to diabetes involved revising the dementia QIC intervention manual to account for:

- Differences in the clinical topic
- NDA contractual requirements
- External context
- Recent evidence [31]

The adapted content and delivery was discussed with the NDA quality improvement team, the NDA experts by experience group and the NDA Executive. We revised the manual based upon their feedback.

### Phase 5 results

Table 4 summarises the adaptation work. Virtual delivery provided the opportunity to split the full-day workshop into three two-hour workshops to reduce burden and create opportunity for participating teams to consider the content between sessions. The revised intervention was presented to the NDA experts by experience group and the NDA Executive, both of which supported the proposed approach.

**Table 4:** A summary of the aim, methods and results for each intervention development phase

Phase and aim	Methods	Key findings
Phase 1: To describe the response to a national audit	Interviews, observations and documentary analysis, iteratively presenting the findings to both a co-design group and an advisory group	Data collection was manual, took an average of 37 min per record and involved variable interpretation both of the notes and the standards being measured. Feedback took 15 months and was typically seen by two or three people. The clinical lead was responsible for developing an organisational action plan. They did this drawing upon national, rather than local, results. The actions were constrained by what the clinical lead could personally deliver. There was little evidence that they selected actions aligned to an analysis of influences upon performance. The action plan was reviewed, amended and approved at directorate- and organisational-level committees. In reaching their decision, the committees discussed the motivation of the audit provider, the validity of the data, relative performance, triangulation with other data and risks to organisational priorities.
Phase 2: To identify and specify enhancements	Co-design methods involving facilitated discussion about evidence, theory and the use of a specifying framework. Research team developed a logic model to review theoretical coherence.	The co-design group identified the opportunity to enhance data collection, feedback and action planning. They prioritised action planning and defined the outcome sought. The research team identified theoretically-coherent target behaviours that aligned to the outcome: target low baseline performance; address recipient priorities; develop trust and credibility; present meaningful comparisons; present loss-framed data; identify and address barriers to performance; develop a conceptual model; involve stakeholders; consider the opportunity cost. The co-design group considered these proposals, rejecting the use of loss-framing. The co-design group specified how the selected behaviours could be delivered. Their specification was used to group the target behaviours into a series of ‘steps’ (Table 1).
Phase 3: To develop a strategy to implement the enhancements	Co-design methods involving facilitated discussion using theory-informed toolkit	The key normalisation process theory mechanisms anticipated to influence implementation were coherence and cognitive participation. Individual and collective specification, initiation, legitimation were important ingredients. There were differences in the ingredients between the steps. We selected a strategy including an educational workshop and virtual educational outreach. Figure 2 represents the content of the intervention after phase 6; at phase 3, the target behaviours did not include collaboration nor feedback.
Phase 4: To test and refine the intervention	Delivery of the intervention followed by interviews exploring fidelity, feasibility, acceptability and appropriateness [9, 23, 27]	All the BCTs in the manual were delivered. There was evidence for fidelity of receipt, fidelity of enactment and of acceptability of the intervention. Potential enhancements to the intervention included addressing the time commitment, creating opportunities to learn from others, further supporting the analysis of influences and developing content so those developing improvement actions gain feedback about the impact of their actions. We revised the content, re-named the intervention, amended activities to address time commitment, incorporate a more structured analysis of influences, support collaboration and to develop local feedback mechanisms.
Phase 5: To adapt the intervention to a second national audit	Re-design and consultation to reflect differences in clinical topic, context, contractual requirements and recent evidence.	Delivery changed in response to: the new clinical topic, by changing examples based on dementia standards to those within the diabetes audit and incorporating delivery through a credible source (NDA Clinical lead); context, pandemic related restrictions meant that it was delivered virtually, requiring additional content about the use of Microsoft Teams and Google JamBoard; the NDA contractual requirements, to address a pre-determined target for improvement thereby narrowing the recipients’ selection of priorities (step 1); recent evidence [26], that there may be benefits from highlighting the intervention deliverers’ recent and credible experience in quality improvement and having multi-disciplinary quality improvement team delivery that includes opinion leaders.
Phase 6: To test and refine the intervention	Coding the BCTs within the manual, delivery of the intervention followed by coding the BCTs delivered and interviews exploring fidelity, appropriateness and acceptability. Re-design and consultation to reflect interview findings.	All BCTs identified in the written protocol were delivered by facilitators. Participants reported positive attitudes towards the intervention and that the intervention was appropriate.

**Fig. 2:**
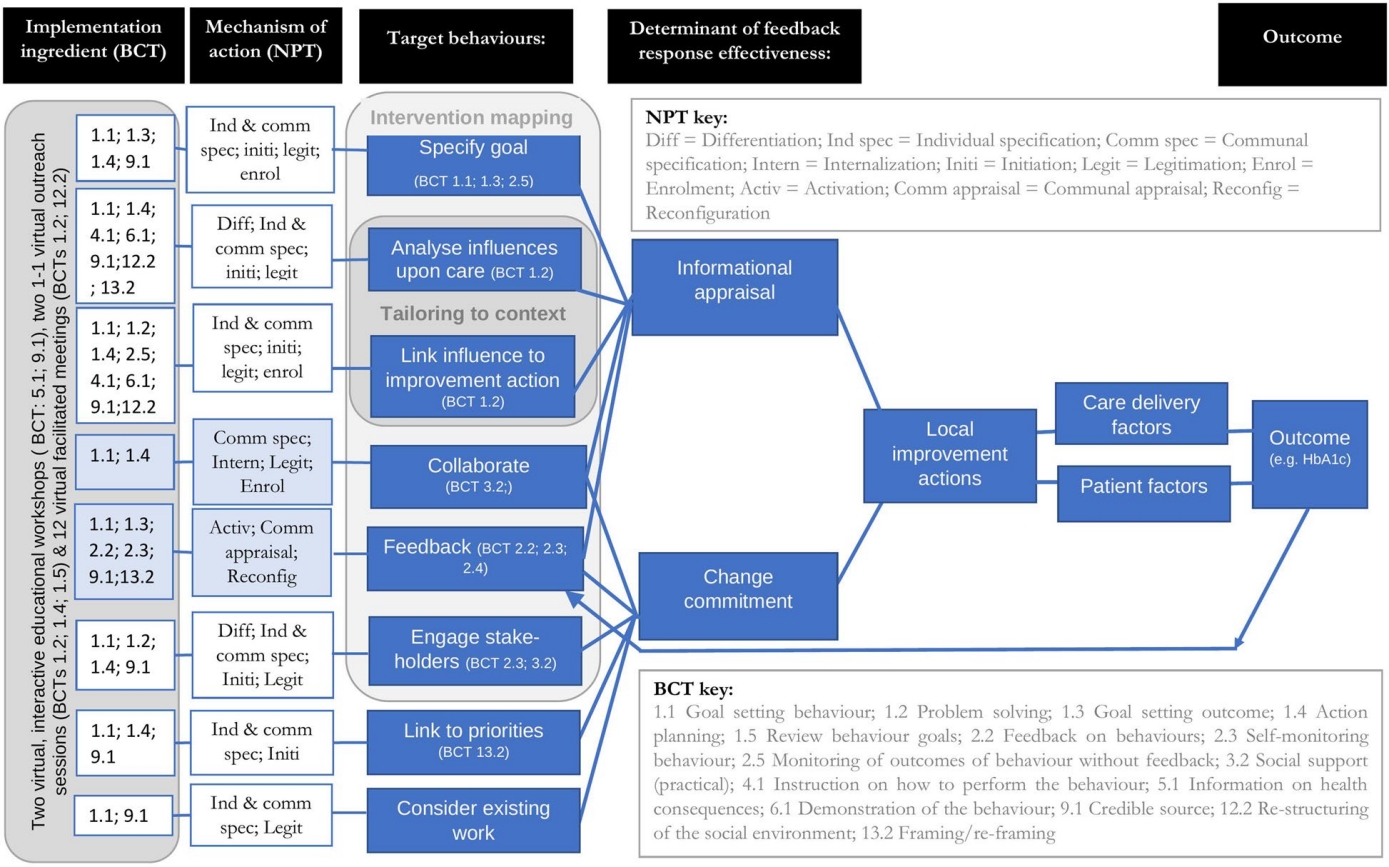
Intervention logic model after phase 6

### Phase 6 method

The aim for phase 6 was to refine the intervention based upon a further feasibility study exploring fidelity of delivery, appropriateness and acceptability of the intervention. The work was undertaken by researchers independent of the intervention development team (LM, EOH and JMc). To evaluate fidelity of delivery, the intervention manual and materials were coded for behaviour change techniques (BCTs) [12] and compared to the BCTs delivered during the online delivery of the workshop. Approximately 10% of the materials were double coded by EOH and LM and intercoder reliability was calculated. The intervention was delivered by the multidisciplinary NDA team (NDA Clinical lead, previous 2017–2019 QIC participants, and facilitator (MS)) to two cohorts: one targeting improvement in type I diabetes; and one targeting improvement in Type II diabetes. Delivery of both type I and type II interventions was recorded and coded (EOH identified the BCTs delivered in the type I intervention; LM identified the BCTs delivered in the type II intervention). Coding discrepancies were discussed with a third author (JMc) in order resolve disagreements. The percentage of BCTs delivered as specified in the manual and materials for both type I and type II diabetes was calculated. Semi-structured interviews with intervention recipients explored components of the theoretical framework of acceptability [32]. All recipients were invited to be interviewed. Thematic analysis [27] was used to analyse the interview data. The findings were presented to the research team who proposed refinements. The design of the refined collaborative, and examples of the work being undertaken by participants, was presented to the NDA Experts by experience group and the NDA Executive. The manual was further revised based upon this discussion.

### Phase 6 results

The intervention was delivered to 17 teams from across England and Wales, as part of two cohorts focussing on: reduction in team median HbA1c in people with type I diabetes (10 teams, observed by EOH); reduction of cardio-vascular risk in people with type II diabetes (7 teams, observed by LM). The BCTs identified in the manual were delivered to each cohort (Table 5). There was 83% agreement on the initial double coding of BCTs identified in intervention manuals and materials. The review of the intervention manual identified twelve BCTs. Table 5 describes the BCTs identified in the manual and within intervention delivery.

**Table 5:** BCTs observed in written materials and intervention webinars

Identified in written materials	Delivered
1.1 Goal setting (behaviour)	Yes
1.2 Problem solving	Yes
1.3 Goal setting (outcome)	Yes
1.4 Action planning	Yes
2.2 Feedback on behaviour	Yes
2.3 Self-monitoring of behaviour	Yes
2.3 Self-monitoring of outcome	Yes
2.5 Monitoring of outcome of behaviour without feedback	Yes
5.1 Information about health consequences	Yes
9.1 Credible source	Yes
12.2 Re-structuring of the social environment	Yes
13.2 Framing/re-framing	Yes

There were two minor losses of fidelity: a number of BCTs intended to be delivered in session 1 were, instead, delivered in session 2, due to time constraints; and BCTs present in the manual were occasionally delivered at a different time from when indicated, albeit within the same workshop. Five healthcare professional intervention recipients were interviewed. Interviewees described that the intervention was acceptable and appropriate, describing a positive affective attitude and that the burden may be worth the opportunity cost (Table 6).

**Table 6:** Example quotes from phase 6 feasibility study

**Acceptability**	**Affective attitude and opportunity cost** “I think it’s been really lovely...I’m really quite enjoying it” “the programme has been really, really good. I feel like, you know, there’s been some brilliant opportunities from it”
	**Perceived effectiveness** “we are already beginning to see it” “Yes, I do think attending them will hopefully be helpful to the success of our initiatives”
	**Burden** The QIC was time-consuming and required some effort, but was worthwhile: “we don’t get additional time or resources to do it. So, at this point, it’s your own goodwill that you are doing the extra work… I’m really quite enjoying it, and we are already beginning to see the improvements” (Interviewee 4). Part of the perceived burden reflected the current context: “At the moment, it’s particularly tricky for everybody who’s working in the NHS with the pandemic and now with recovering...”
**Appropriateness**	“I thought it was managed really, really well. I don’t feel like I’ve missed out with it being virtual, I think it worked well”. (Interviewee 3) [Participants] “have a chance to raise a hand [referring to the ‘raise hand’ function in Microsoft Teams] and bring their point forward so, you get a more balanced view” (Interviewee 6). Participants described that virtual delivery increased accessibility and made the intervention more easily incorporated into busy clinical schedules, but may have led to a loss of informal sharing of learning, for example over coffee breaks.

In reviewing the content and work of the NDA QIC, the NDA *Experts by experience* group were supportive of the current design. They asked for increased content to support teams to engage with local service users. Intervention content in relation to stakeholder engagement was extended in response to their feedback. The intervention is described in the TIDieR checklist (Table 9 in Appendix 4).

## Discussion

We undertook multiphase development of an intervention to enhance national audit. In phase 1, we used multiple qualitative methods to describe what happens when a national audit reaches the hospital. Phases 2 and 3 used co-design methods to select the target for enhancement and subsequently to specify an intervention to implement the enhanced action planning process. In phase 4, we explored the intervention as an adjunct to the national audit of dementia and subsequently refined it. In phase 5, we adapted the intervention to a different audit, the national diabetes audit. Phase 6 involved a second exploration of the intervention, as an adjunct to the national diabetes audit, and led to further refinement. The resultant intervention is a specified national audit quality improvement collaborative involving virtual workshops, virtual outreach and virtual facilitated collaborative meetings led by a multidisciplinary team able to deliver the BCT ‘credible source’.

Complex interventions may be flexible [8]. The QIC intervention has been manualised to support fidelity of delivery across deliverers and been tested through both face-to-face and virtual delivery. The intervention supports tailoring, whereby participants are supported to analyse their local context using the Theoretical domains framework and to select improvement strategies aligned to their analysis. The analysis of influences happens formally within one exercise started during the second virtual workshop, whereas the local context may be dynamic (e.g. [33]). Introduction of the feedback mechanism and on-going facilitated virtual meetings provide a prompt to address emergent contextual influences. This tailoring work is proposed to support the intervention to be applicable across contexts, a proposition that will be explored further in the future process evaluation.

Across our intervention development Phases, and within quality improvement work (e.g. [34]), the issue of time is an important consideration. In phase 3, the exploration of influences upon implementation identified that clinicians leading the hospital response to the national audit may not have the time to undertake the identified quality improvement practices. The co-design group proposed this might be addressed by changing those undertaking the work. A further suggestion by both the co-design group and participants in phase 4 was for the clinical lead to negotiate time with the clinical director, although phase 4 participants indicated this might be more to gain recognition of the time costs rather than actually having time released for the work. Phase 4 participants described that the burden of the intervention may be worthwhile and a suggestion that their personal goals influenced their assessment of the intervention.

To address the time burden, we provide potential participants with information about the time costs during recruitment and considered which tasks could be undertaken by different actors not currently involved in the response to national audit, specifically the organisational improvement team to undertake observations, librarian to undertake systematic reviews or clinical governance team to identify priorities described in organisational documents. The workshops duration was extended so that enacting the QIC practices (e.g. specify goal, identify stakeholders) was undertaken within protected education time. The intervention includes content both to implement the negotiation of time through job plans and to influence participants’ interpretation of the burden through previous QIC participants describing that improvements make the time worthwhile. Previous work has found quality improvement collaboratives to be cost-effective [35]. Future work will investigate the cost-effectiveness of the National Audit QIC.

The work in phase 6 to code BCTs within the intervention highlighted the need to distinguish between active ingredients at different levels, for example:

- The target behaviours being implemented as part of the enhanced local improvement work, for example, specifying the aim for the improvement delivers the BCT goal setting (outcome)
- The BCTs delivered in order to implement the new behaviours (e.g. restructuring the social environment to analyse influences upon care)
- The BCTs that support acceptability of the intervention (e.g. credible source giving information about information about health consequences to increase buy-in to the intervention).

## Strengths and limitations

This paper describes iterative intervention development that draws upon evidence, theory, stakeholder views, gives specific consideration of implementation and explores the feasibility in two contexts. Intervention development has been described in line with guidance (Table 9 in Appendix 4). The work exemplifies multi-purpose application of theory: the articulation of the programme theory as a logic model served to support exploration about how the intervention may influence outcome. To develop the intervention content, we drew upon earlier theory-informed proposals describing influences upon the effectiveness of audit and feedback [20]. To review coherence, we considered the logic model in the context of earlier theory. To explore influences upon implementation, we used a theory-informed toolkit [19].

There are limitations to the work. The first feasibility study, and to a lesser extent, the second feasibility study, faced challenges recruiting interview participants. It is anticipated that this reflected participant availability during the pandemic, and perhaps also that participants were undertaking additional work as a result of the intervention. Participant responses point towards the acceptability, appropriateness and feasibility of the intervention, initial findings which will be built upon alongside later work to test effectiveness. The design of the process evaluation will take into account an evaluability assessment [8] by placing greater emphasis on methods with lower participant burden (observation and documentary analysis). The method, illustrated in Fig. 1, suggests a linear process. In reality, there were feedback loops, for example, the phase 3 work to identify influences upon implementation led to the phase 2 content being revisited (e.g. to remove loss-framing). Similarly, it is anticipated that the logic model illustrates the stronger relationships between components, but it is likely that there are lesser interactional effects; for example, cognitive participation in one target behaviour may influence buy-in to another, consideration of existing work may influence both the assessment of opportunity cost proposed to affect change commitment and the informational appraisal of implementation capability. The iterative development process has developed and refined the intervention, it is anticipated that the intervention will be refined further through later learning.

The intervention is a quality improvement collaborative, containing educational workshop and outreach strategy for local team leads and facilitation of a learning collaborative. Quality improvement collaboratives are a common method to improve healthcare [31]. There is evidence that they may be effective, but a lack of justification for the content, incomplete reporting and multiple sources of bias undermine interpretation of the results [36, 37]. A strength of the current paper is the description from the selection of the target for enhancement that built upon inductively developed description of current process, through to a coherent, specified, manualised and feasible intervention.

## Conclusion

We undertook iterative co-design work, building upon inductively identified opportunities to enhance national audit through the systematic use of theory-informed proposals, evidence and stakeholder input to develop an intervention. We explored the feasibility of the intervention as an adjunct to two national audits. The intervention seeks to increase feedback recipients’ quality improvement capabilities by implementing target behaviours consistent with the organisational readiness to change theory [23], such that local teams tailor improvement actions to their local context and develop organisational commitment. We plan to evaluate the effectiveness of the intervention as part of a cluster randomised trial and process evaluation. The planned study will investigate whether NDA plus the National Audit QIC leads to greater improvement in patient outcomes compared to NDA feedback alone. The theory-informed process evaluation will explore diabetes specialist teams’ engagement, implementation, fidelity and tailoring. The economic evaluation will micro-cost the QIC, estimate cost-effectiveness of NDA feedback with QIC and estimate the budget impact of NHS-wide QIC roll out. Further planned work will explore adaptation through work to adapt the intervention to three further audits in a different national context.

## Data Availability

The datasets generated during and/or analysed during the current study are not publicly available in order to maintain the anonymity of participants.

## Appendix 1

Table 7

**Table 7:** Co-design group definitions to terms within the initial outcome of the intervention

Term	Co-production group definition
‘Target poor performance’	Poor performance should be defined by each recipient in both absolute terms and by considering performance relative to other hospitals (e.g. lower quartile).
‘Relevant’	Recipients understand and the actions address the reasons for poor performance.
‘Actionable’	Action is resourced and agreed.
‘Specific’	States who would be doing what, as part of the action to improve care.
‘Time-bound’	When the action to improve would be completed.
‘Measurable’	How completion of the action will be confirmed.

## Appendix 2

### Summarised notes made during phase 4 intervention delivery

During the workshop activity to analyse performance participants explored the impact of data collection process upon performance, having someone involved in data collection in the workshop was valuable;

Participants jumped to solutions and stakeholders associated with solutions, rather than specifying the behaviour before identifying stakeholders and exploring influences;

Negotiating organisational rules about the use of comparators was important; the term ‘cognitive load’ appeared confusing;

Participants drew on memory to triangulate, a prompt to check their memory against data may be useful;

The exercise to review potential strategies from the adapted ERIC list [22] reduced group energy and appeared burdensome;

The logic model exercise appeared both difficult, but valued, by participants;

The workbook used within the workshop was observed to be a valuable support for the graded tasks and provided a reminder after the workshop, but both observation and interviews identified the opportunity to combine exercises and move others to whole group exercises.

## Appendix 3

Table 8

**Table 8:** GUIDED checklist (Duncan et al. 2020) [10]

1. Report the context for which the intervention was developed.	Background: Variably effective national audits
2. Report the purpose of the intervention development process	Background: “to enhance the effectiveness of national audit through the iterative integration of evidence, theory and stakeholder input”
3. Report the target population for the intervention development process	Methods—phase 4
4. Report how any published intervention development approach contributed to the development process	Background
5. Report how evidence from different sources informed the intervention development process	Method, including Fig. 1
6. Report how/if published theory informed the intervention development process.	Method
7. Report any use of components from an existing intervention in the current intervention development process	Method (phase 5)
8. Report any guiding principles, people or factors that were prioritised when making decisions during the intervention development process.	Method: co-design
9. Report how stakeholders contributed to the intervention development process.	Method and discussion
10. Report how the intervention changed in content and format from the start of the intervention development process	Results
11. Report any changes to interventions required or likely to be required for subgroups	Results: phase 5
12. Report important uncertainties at the end of the intervention development process	Discussion
13. Follow TIDieR guidance when describing the developed intervention	Table 9 in Appendix 4
14. Report the intervention development process in an open access format.	Open access publication sought

## Appendix 4

Table 9

**Table 9:** The TIDieR (Template for Intervention Description and Replication) Checklist

Item number			Where located
**1.**	**BRIEF NAME** Provide the name or a phrase that describes the intervention.	National Audit Quality Improvement Collaborative	9
**2.**	**WHY** Describe any rationale, theory, or goal of the elements essential to the intervention.	the development of commitment and informational appraisal to select actions resonated with the theory of organisational readiness for change (Weiner 2009) [23]	12
**3.**	**WHAT** Materials: Describe any physical or informational materials used in the intervention, including those provided to participants or used in intervention delivery or in training of intervention providers.	The workshop included slides to increase the coherence and cognitive participation of the target behaviours described in the logic model. These were supported by online materials to support participants to identify influences upon participation using the Theoretical Domains Framework, align these influences to actions and to identify stakeholder influence and interest.	13
**4.**	**WHAT** Procedures: Describe each of the procedures, activities, and/or processes used in the intervention, including any enabling or support activities.	The active ingredients are described in the logic model	15
**5.**	**WHO PROVIDED** For each category of intervention provider (e.g. psychologist, nursing assistant), describe their expertise, background and any specific training given.	Facilitator (MS), Clinical Lead, Previous QIC leads	19
**6.**	**HOW** Describe the modes of delivery (e.g. face-to-face or by some other mechanism, such as internet or telephone) of the intervention and whether it was provided individually or in a group.	Virtual delivery through MS teams and using Google JamBoard	19
**7.**	**WHERE** Describe the type(s) of location(s) where the intervention occurred, including any necessary infrastructure or relevant features.	Virtual delivery through MS teams and using Google JamBoard	19
**8.**	**WHEN and HOW MUCH** Describe the number of times the intervention was delivered and over what period of time including the number of sessions, their schedule, and their duration, intensity or dose.	Two virtual workshops, two virtual outreach sessions and 12 facilitated, virtual meetings.	15 and 19
**9.**	**TAILORING** If the intervention was planned to be personalised, titrated or adapted, then describe what, why, when, and how.	Not applicable Note: Tailoring work is undertaken by intervention participants	

## Abbreviations

BCT: Behaviour Change Technique
HbA1c: haemoglobin A1c or glycated haemoglobin, a measure of blood glucose levels
NDA: National Diabetes Audit
NPT: Normalisation Process Theory
PDSA: Plan, do, study, act
ERIC: Expert recommendations for implementing change
QIC: Quality Improvement Collaborative
TIDieR: Template for intervention description and replication
4AT: Rapid clinical test for delirium
